# Effects of Virtual Reality–Based Multimodal Audio-Tactile Cueing in Patients With Spatial Attention Deficits: Pilot Usability Study

**DOI:** 10.2196/34884

**Published:** 2022-05-25

**Authors:** Samuel Elia Johannes Knobel, Brigitte Charlotte Kaufmann, Nora Geiser, Stephan Moreno Gerber, René M Müri, Tobias Nef, Thomas Nyffeler, Dario Cazzoli

**Affiliations:** 1 Gerontechnology & Rehabilitation Group University of Bern Bern Switzerland; 2 Sorbonne Université Institut du Cerveau - Paris Brain Institute (ICM), Inserm Centre national de la recherche scientifique, Hôpital de la Pitié-Salpêtrière Paris France; 3 Neurocenter Luzerner Kantonsspital Luzern Switzerland; 4 Perception and Eye Movement Laboratory Departments of Neurology and BioMedical Research Inselspital, Bern University Hospital Bern Switzerland; 5 Department of Neurology Inselspital, Bern University Hospital University of Bern Bern Switzerland; 6 ARTORG Center for Biomedical Engineering Research University of Bern Bern Switzerland; 7 Institute of Psychology University of Bern Bern Switzerland

**Keywords:** virtual reality, search task, stroke, neglect, multimodal cueing, bird search task

## Abstract

**Background:**

Virtual reality (VR) devices are increasingly being used in medicine and other areas for a broad spectrum of applications. One of the possible applications of VR involves the creation of an environment manipulated in a way that helps patients with disturbances in the spatial allocation of visual attention (so-called hemispatial neglect). One approach to ameliorate neglect is to apply cross-modal cues (ie, cues in sensory modalities other than the visual one, eg, auditory and tactile) to guide visual attention toward the neglected space. So far, no study has investigated the effects of audio-tactile cues in VR on the spatial deployment of visual attention in neglect patients.

**Objective:**

This pilot study aimed to investigate the feasibility and usability of multimodal (audio-tactile) cueing, as implemented in a 3D VR setting, in patients with neglect, and obtain preliminary results concerning the effects of different types of cues on visual attention allocation compared with noncued conditions.

**Methods:**

Patients were placed in a virtual environment using a head-mounted display (HMD). The inlay of the HMD was equipped to deliver tactile feedback to the forehead. The task was to find and flag appearing birds. The birds could appear at 4 different presentation angles (lateral and paracentral on the left and right sides), and with (auditory, tactile, or audio-tactile cue) or without (no cue) a spatially meaningful cue. The task usability and feasibility, and 2 simple in-task measures (performance and early orientation) were assessed in 12 right-hemispheric stroke patients with neglect (5 with and 7 without additional somatosensory impairment).

**Results:**

The new VR setup showed high usability (mean score 10.2, SD 1.85; maximum score 12) and no relevant side effects (mean score 0.833, SD 0.834; maximum score 21). A repeated measures ANOVA on task performance data, with presentation angle, cue type, and group as factors, revealed a significant main effect of cue type (*F*_30,3_=9.863; *P*<.001) and a significant 3-way interaction (*F*_90,9_=2.057; *P*=.04). Post-hoc analyses revealed that among patients without somatosensory impairment, any cue led to better performance compared with no cue, for targets on the left side, and audio-tactile cues did not seem to have additive effects. Among patients with somatosensory impairment, performance was better with both auditory and audio-tactile cueing than with no cue, at every presentation angle; conversely, tactile cueing alone had no significant effect at any presentation angle. Analysis of early orientation data showed that any type of cue triggered better orientation in both groups for lateral presentation angles, possibly reflecting an early alerting effect.

**Conclusions:**

Overall, audio-tactile cueing seems to be a promising method to guide patient attention. For instance, in the future, it could be used as an add-on method that supports attentional orientation during established therapeutic approaches.

## Introduction

Virtual reality (VR) devices are being increasingly used, and can be found in industries [1,2] and areas of entertainment, military [3,4], and medicine [5-11]. Skills learned in VR have been shown to be transferable to the real world [7,12,13], leading to a broad spectrum of possibilities, from training to rehabilitation. Virtual environments have the advantage of being fully customizable, and therefore, they are potentially adaptable to different situations and even different user abilities [14,15]. Particularly in the field of rehabilitation of neurological disorders, VR has been shown to be a promising approach because of this adaptability and the inclusion of different sensory information [16]. This is believed to not only promote cortical reorganization but also facilitate the activation of neuronal plasticity [17]. One of the possible applications of VR in this area is the creation of an environment that can be manipulated in a way that would help patients with disturbances in the spatial allocation of visual attention. These disturbances are often subsumed under the label of visual neglect, a frequent condition occurring after right hemispheric stroke (up to 70%) [18,19]. Visual neglect is characterized by the inability to respond or react to targets coming from the contralesional side of space. It is a negative prognostic factor for the overall outcome after stroke and is difficult to treat [20].

One approach to ameliorate visual neglect is to apply cross-modal cues (ie, cues in sensory modalities other than the visual one) in order to guide visual attention toward the neglected side. Most commonly, auditory cues have been successfully applied in neglect patients in order to guide visual attention toward the contralesional side [21-24]. Although less often investigated, tactile cues (alone or in combination with auditory cues) seem also to be able to ameliorate the contralesional allocation of visual attention in this patient population [21,25].

Most setups of the above-mentioned studies were based on 2D screens, complex speaker arrays, or loudspeakers on moving robot arms. The use of a 3D VR environment can reduce the complexity of the system and increase its ecological validity, owing to the higher immersion, larger visual field, and possibility to freely move the head and body [26].

Indeed, the effects of auditory cues, presented in a VR setting, on visuospatial attention deployment have been successfully explored in neglect patients and show promising results [27,28]. In contrast, to the best of our knowledge, no study has so far investigated the effects of tactile cues in VR on the spatial deployment of visual attention in neglect patients, although results obtained in healthy controls seem encouraging [29,30]. Finally, previous results in 2D settings suggest that multimodal cueing (ie, combining auditory and tactile cueing at the same time) may result in superior effects than single cueing [29,31]. However, the effects of this combination for patients with visual attention deficits in a 3D VR setting have not been studied.

Thus, this pilot study aimed to investigate the feasibility and usability of multimodal (auditory and tactile) cueing, as implemented in a 3D VR setting (our bird search task), in patients with visual neglect, as well as obtain results concerning the effects of different types of cues on visual attention allocation compared with noncued conditions in these patients. We hypothesized that (1) the implementation of a new system, including tactile, auditory, and combined audio-tactile cueing, is feasible and usable for patients with impaired spatial attention; (2) the different cue types have a positive effect on the visuospatial attention allocation ability of patients; and (3) the use of multimodal cues (combined auditory and tactile) has larger effects than unimodal cues (auditory or tactile alone).

## Methods

### Demographics

Between June 2020 and November 2020, 12 patients with left-sided visual neglect after subacute right hemispheric stroke were recruited and included in the study.

**Figure 1:**
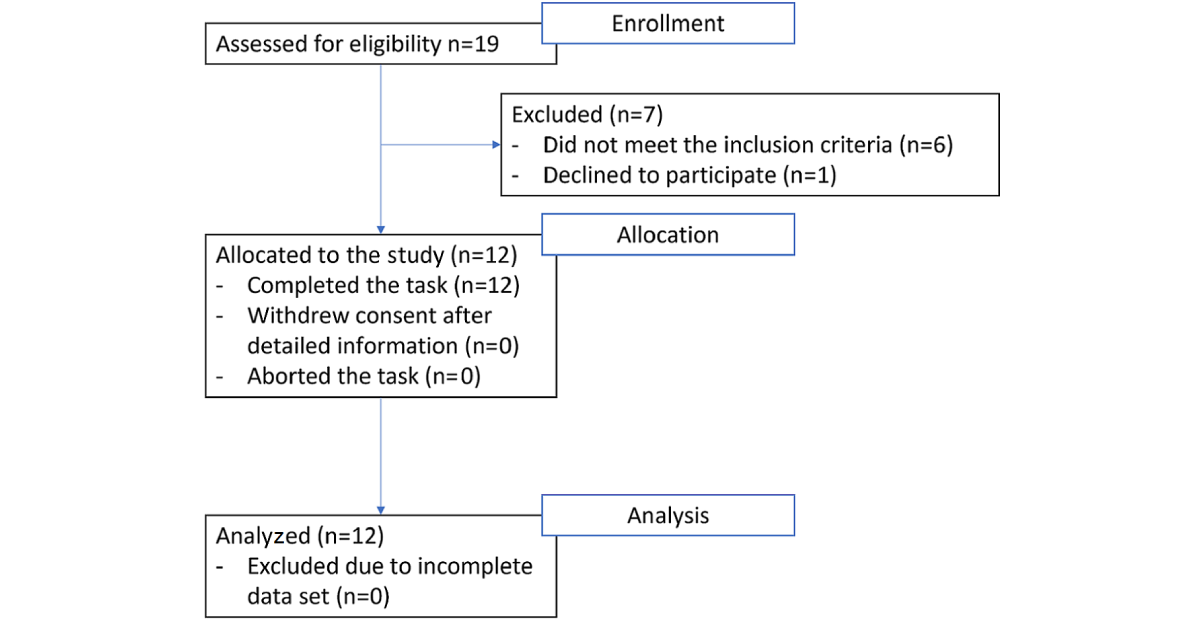
Patient flowchart from enrollment to analysis.

They were inpatients at the Neurorehabilitation Clinics of the Inselspital, Bern University Hospital, or the Kantonsspital Luzern, Switzerland. All patients had normal or corrected-to-normal vision. The mean age of the patients was 58.2 years (SD 9.70 years), and 4 were female. See Table 1 for detailed information and Figure 1 for the recruitment flowchart.

**Table 1:** Detailed demographics and results of the neuropsychological tests of the participants.

Patient ID	Gender	Age (years)	CBS^a^ score	CoC^b^	LBT^c^	Somatosensory impairment
P01	Male	54	8	0.084	12.68%	Yes
P02	Male	42	4	0.043	4.98%	No
P03	Female	63	4	−0.107	5.28%	No
P04	Female	63	10	0.490	65.57%	No
P05	Male	46	8	0.024	11.11%	No
P06	Male	61	15	0.699	5.75%	No
P07	Female	66	4	0.003	2.70%	Yes
P08	Male	69	13	−0.005	9.34%	No
P09	Female	44	9	−0.004	1.24%	Yes
P10	Male	58	12	0.863	37.13%	Yes
P11	Male	61	14	0.739	3.32%	Yes
P12	Male	71	19	0.740	3.81%	No

^a^CBS: Catherine Bergego Scale [32].

^b^CoC: center of cancellation [33].

^c^LBT: Line Bisection Text (relative deviation in % [34]).

The inclusion criterion was a pathological score in 1 of the following 3 assessments: Catherine Bergego Scale (CBS) [32], Sensitive Neglect Test (SNT) [35], and Line Bisection Test [34].

The CBS is an established questionnaire allowing to detect the presence and severity of neglect based on the observation of everyday life activities (cutoff for neglect: CBS score >1). The SNT is a paper-pencil cancellation task. The spatial distribution and number of missed targets are evaluated using the center of cancellation (CoC; cutoff: CoC >0.081) [33]. The CoC reflects the mean position from the center to the missed targets and is normalized to values from −1 to 1. Zero indicates no spatial bias, negative values indicate a shift towards the left, and positive values indicate a shift towards the right. Furthermore, the Line Bisection Test [34] is another frequently used neuropsychological task to assess neglect severity, with a cutoff value of ≥11% mean relative rightward deviation [34].

Somatosensory impairment, and hearing and auditory extinction were assessed clinically. Somatosensory impairment was assessed by comparing the sensitivity for touch on the forehead and the temporal head region between the left and right. To assess hearing and auditory extinction, rustling was presented to each ear individually or to both ears simultaneously, and patients’ reports were compared. Based on this assessment, patients were assigned to the following 2 subgroups: patients with and without sensory impairment. As no patient had auditory extinction, no auditory extinction group was formed.

### Ethics Approval

The study was approved by the Ethics Committee of the Cantons of Bern and Lucerne (ID 2017-02195), and was conducted in accordance with the latest version of the Declaration of Helsinki. All participants signed written informed consent forms before participation.

### Questionnaires

A selection of different questionnaires was administered to assess the usability and side effects of the VR task, as previously described by Gerber et al [36,37].

For assessments of acceptance, usability, and participant perception of the visual search task and the VR system, the System Usability Scale (SUS) [38] was used. To assess side effects and cybersickness [39], the Simulator Sickness Questionnaire (SSQ) [40] was used.

### Technical Setup

The VR hardware consisted of a stand-alone head-mounted display (HMD) and a hand-held controller. During the task, the positions of the controller and the HMD were continuously recorded (20 Hz sample rate).

The resolution of the HMD (Oculus Quest, Facebook Technologies) was 1440×1600 pixels, with a horizontal field of view of 110 degrees and a frame rate of 72 Hz. For the development of our bird search task, the platform Unity [41] was used.

To monitor the patient’s behavior during the task, the patient’s view was projected to a laptop computer using a wireless streaming tool via Sidequest [42]. The experimenter was thus able to see what the patient saw in real-time and could intervene if needed.

Additionally, for the application of the tactile cue, a special cushion as an inlay piece for the HMD was used, as described by Knobel et al [29]. The cushion contains 6 coin vibrators symmetrically distributed over the forehead. Each coin vibrator could be started individually. The sound was presented via over-ear headphones.

### Bird Search Task and Study Procedure

**Figure 2:**
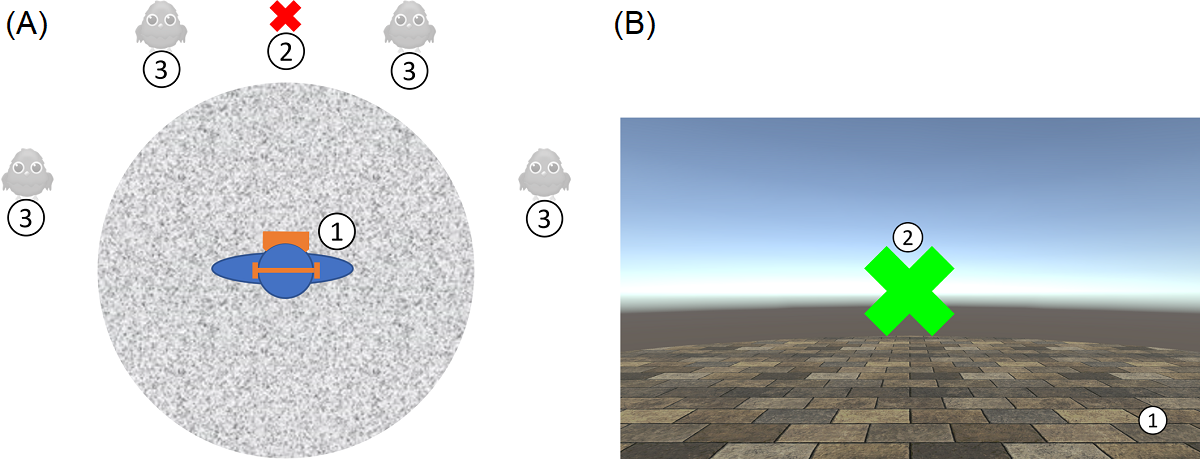
(A) Scenario setup in the virtual environment, with the following 3 main features: (1) An empty floor, on which the player was positioned at the center of the scene; (2) A central fixation cross; and (3) Four presentation angles (−70°, −30°, 30°, and 70°) where the birds could appear in the bird search task. (B) Patients view when starting the task with the floor (1) and the central fixation cross (2).

The main goal of the bird search task was to detect the appearing birds as quickly as possible. The task took place in a virtual environment and contained 3 main features (Figure 2).

First, there was a plane surface, looking like a floor, which prevented the patient from feeling like floating or even falling. Second, directly in front of the patient, a colored fixation cross was presented. The patient’s initial position was aligned, so the fixation cross was straight ahead. Third, there were 4 presentation angles at which the stimuli (blue birds), one at a time, could appear. These presentation angles were on a horizontal line at −70°, −30°, 30°, and 70°, as defined with respect to the patient’s trunk (see the presentation angles of 4 birds in Figure 2). In this study, we will consider the 30° and −30° angles as paracentral angles and the 70° and −70° angles as lateral angles.

**Figure 3:**
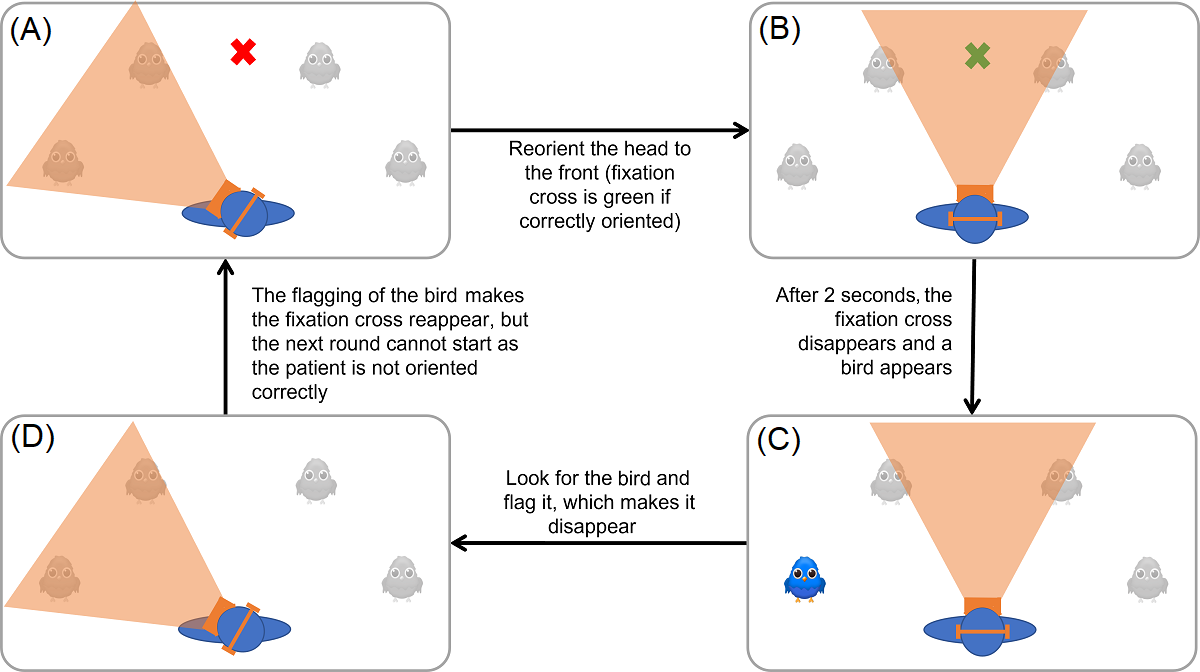
Schematic representation of an exemplary trial of the bird search task. The grey birds represent the potential presentation angles, where the birds could appear. (A) The patient’s head (in blue) is not oriented toward the fixation cross, and therefore the task cannot start. (B) After reorienting the head toward the fixation cross, the cross turns green. (C) After 2 seconds of being correctly oriented, the fixation cross disappears and the bird appears (in this case, the bird appears at the presentation angle −70°). (D) The patient orients toward the bird and flags it, so it disappears. This results in the reappearance of the fixation cross that stays red as long as the patient has not reoriented toward it.

At the beginning of each trial (a full round in Figure 3), the patients were asked to orient the HMD toward a central fixation cross (red cross in Figure 2; Figure 3A), thereby aligning their head to the trunk straight ahead. The fixation cross had the property to turn green if aligned with the HMD, thereby giving feedback for being correctly fixated (Figure 3B). After fixating for 2 seconds on the central fixation cross, it disappeared and, at the same time, a target bird appeared (Figure 3C). Consequently, the disappearance of the central fixation cross was a signal for the patient to start searching for the target bird. The patient was asked to confirm the detection of the target bird by flagging it with a hand-held controller. A flagged bird disappeared from the scene by falling. This started a new trial (ie, central fixation cross for 2 seconds, disappearance of the latter, and concomitant appearance of the next target bird; Figure 3D). If the target bird was not found within 11 seconds, it simply disappeared, and the next trial started.

Each target bird could appear either with or without additional spatial cues. There were 2 different possible spatial cues (auditory or tactile), resulting in the following 4 different conditions: None (no cue), Audio (only auditory cue), Tactile (only tactile cue), and Combo (auditory and tactile cues; for more details, see the “Cues” section below).

In the None condition, the patient had to start looking for the target as soon as the fixation cross disappeared, but no additional hint for the direction was given. In the 3 other conditions, the patient received an additional hint from the presentation angles of the newly appeared target bird at the same time the target appeared. The cues were repeated after 3 seconds until the maximum presentation time of 11 seconds.

The bird search task was organized into 4 sessions, each containing a single spatial cue type (ie, None, Audio, Tactile, and Combo). In each session, 80 target birds were presented (ie, each of the 4 presentation angles were tested 20 times). The order of the presentation angles at which the target birds appeared was random, with the constraint that 2 target birds could not appear at the same presentation angle in 2 consecutive trials. The order of the 4 sessions was randomized. The 4 sessions took place over 2 consecutive days (ie, 2 sessions per day). On the first day, all the conditions were explained to the patient, and 3 practice trials were performed. During the practice trials, each cue type and each presentation angle was presented. First, the patient was verbally guided to the target, and if the position was not detected correctly, the corresponding practice trial was repeated up to 3 times. If the position was detected correctly, the next angle/cue was presented. After completing the practice, the first 2 sessions were performed. On the second day, the corresponding practice trials of the 2 remaining cueing conditions were repeated, and then, the 2 remaining sessions were performed.

### Cues

The task included auditory and tactile spatial cues. The auditory cue was a 1-second tone of 500 Hz with 0.1 seconds fading in and out to reduce the sound’s sharpness. The spatial information of the auditory cue was generated by the audio spatializer software development tool kit that is part of the Unity game development platform [41]. The head-related transfer function of the spatializer is based on the KEMAR data set (set of pre-ear impulse recordings of a dummy head; Bill Garnder at MIT Media Lab [43]). In the task, the sound was realistically perceived as coming from the left (due to different sound volume levels between the 2 ears) if the sound source was on the left side of the head and as coming from the front if the head was oriented toward the sound source.

The tactile cue (1-second vibration) was applied using punctual vibration that came from a special inlay of the HMD that contained 6 symmetrically positioned individually controllable coin vibrators [29]. The coin vibrators were controlled in a way that mimicked the spatial behavior of sound. This means that if the head is oriented to the front and a cue is given on the left side, the most left coin vibrator is activated, signaling that the cue is on the left side. If the head is then turned and faces the cue, the middle coin vibrators are activated, signaling that the cue is now directly in the front of the patient. If the head is turned even more, the right coin vibrators are activated, signaling that the cue is now on the right side.

In the Combo condition, auditory and tactile cues were given simultaneously for 1 second each.

### In-Task Parameters

#### Performance

The main parameters assessed were the mean time until targets were flagged and the percentage of found and flagged birds for each presentation angle and condition. From the percentage found and the mean time of found targets, we calculated a performance measure [44]. The performance measure was calculated for every presentation angle and condition by dividing the percentage of found targets by the mean time needed to flag the targets. A higher value as a result of this calculation thus represents better performance.

This performance measure allows combining both behavioral aspects (ie, accuracy and speed of reactions) within a single parameter. With respect to the cognitive impairments of neglected patients, this performance measure offers several advantages. First, it allows quantifying performance even if a neglect patient does not find any target for a given angle/condition. Indeed, in this case, it would not be possible to calculate the mean time to flag targets. However, as the value is multiplied by the percentage of found targets (in this case, 0%) in the formula, the result would correctly be 0 (indicating very low performance for that particular presentation angle and condition). Second, this performance measure is robust against extreme values.

If, for instance, a patient finds only 1 target in a very fast way in a certain presentation angle/condition combination, this low reaction time would be overrepresented if only the mean time to flag targets is analyzed. Instead, in the performance measure formula, the inclusion of the percentage of found targets reduces the weight of this extreme value [44].

#### Early Orientation

**Figure 4:**
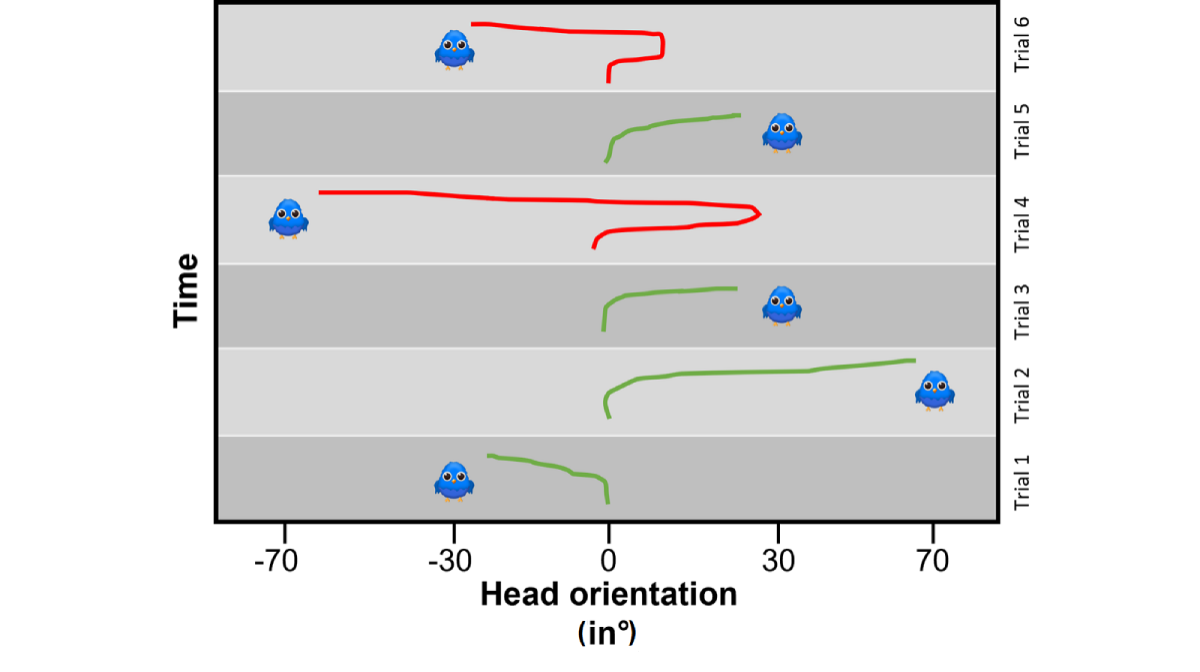
Schematic representation of 7 trials and how the ratio of correct early orientation was calculated. The x-axis shows the head angle from the point where the target appears until it is found. The angle is in relation to the starting position of the trunk. Green represents traces of correct orientation and red represents traces of incorrect orientation.

During the task, the angular position of the HMD with respect to the trunk was continuously recorded, thus reflecting the head rotation over time. Based on these data, the early orientation [45-47], that is, the direction (to the left or right) of the first head rotation movement after the presentation of a stimulus, was extracted. We then used the ratio of correct early orientation instances (ie, the instances in which the head was initially turned to the left even though the target appeared on the right, and vice versa; Figure 4) per condition and presentation angle as an in-game performance parameter.

Figure 4 shows a schematic representation of the early orientation parameter. The traces (green and red lines) represent the head orientation during the presentation of 6 targets (blue birds). In this example, when the fourth and sixth targets were presented (red traces), the patient first turned the head toward the right, even though the target appeared on the left. When the first, second, third, and fifth trials (green traces) were performed, the orientation was correct from the beginning. Thus, the ratio of correct early orientation instances for the right side is 1 (ie, all early orientation instances were correct when the target appeared on the right side) and for the left side is 1/3.

The early orientation was determined by comparing the head’s angle when the target appeared (central) with the head’s angle after 1.5 seconds. If the difference (start angle minus angle after 1.5 seconds) was negative, the patient had turned the head to the right; otherwise, the patient had turned the head to the left.

### Data Collection and Statistical Analyses

Statistical analyses were performed once for all patients together, and comparisons were performed between the with and without somatosensory impairment groups (both groups had no auditory extinction).

First, we analyzed the effects of the different cueing conditions and of the different spatial positions on in-game parameters by means of a 2-way repeated measures ANOVA, with cue type (levels: None, Audio, Tactile, and Combo) and angle (levels: −70°, −30°, 30°, and 70°) as within-subject factors for all participants.

Second, since it is assumed that auditory and tactile cueing require intact somatosensory processing to be effective, we aimed to assess the effect of impairment in the respective modality. For this purpose, we grouped the patients according to somatosensory impairment (yes/no) and auditory extinction (yes/no), and reran the ANOVA with group as a between-subject factor (levels: with and without somatosensory impairment), and cue type (levels: None, Audio, Tactile, and Combo) and angle (levels: −70°, −30°, 30°, and 70°) as within-subject factors.

Post-hoc analyses with least significant difference were applied for the identification of significant differences for cues within presentation angles. The study data were managed using Research Electronic Data Capture [48,49], a web-based tool to support data handling for research studies. Data analyses were performed with R (R Foundation for Statistical Computing) and MATLAB (MathWorks).

## Results

### Subgroups

With regard to the neuropsychological measures, the mean results were as follows: 10.0 (SD 4.79) points for the CBS score [32], 0.30 (SD 0.37) for the CoC [33], and 13.6% (SD 19.0%) relative deviation toward the right for the Line Bisection Test [34].

On separation for somatosensory impairment, there were 7 patients without and 5 with somatosensory impairment of the front head. On comparing age and neuropsychological measures between the groups, there were no significant differences for age (*t*_9.94_=0.480; *P*=.64) and the neuropsychological measures (CBS: *t*_10.0_=0.376, *P*=.72; CoC: *t*_7.78_=−0.289, *P*=.78; Line Bisection Test: *t*_9.99_=0.343, *P*=.74).

### Questionnaires

Usability (based on the SUS ratings) was rated with the sum of 3 questions (responses ranging from 0 [unusable] to 4 [highly usable]) and reached a value of 10.2 (SD 1.85) out of a maximum of 12. Both groups provided similar ratings, with no significant difference between the groups (*t*_8.95_=−1.54; *P*=.16).

The occurrence of side effects was assessed with the sum of 7 frequent items from the SSQ (responses ranging from 0 [no side effects] to 3 [severe side effects]), and the score was 0.833 (SD 0.834) out of a theoretical maximum of 21, indicating a very low rate of side effects. The results were not significantly different between the groups (SSQ: *t*_9.86_=−1.77; *P*=.11).

### In-Task Parameters

#### Performance: All Patients

**Figure 5:**
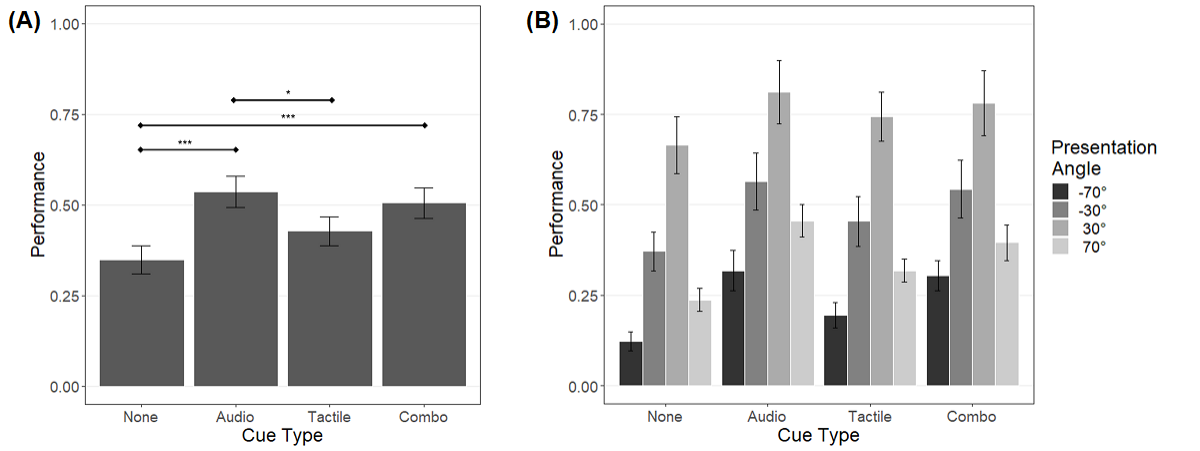
(A) Performance per cue type for all patients (n=12). The whiskers represent the standard error of means. The asterisks represent the level of significance of post-hoc tests (*P<.05, **P<.01, ***P<.001). (B) Visualization of the performance per cue type and presentation angle.

Results concerning all patients are shown in Figure 5. Two-way repeated measures ANOVA showed significant main effects of cue type (*F*_33,3_=7.60; *P*<.001) and presentation angle (*F*_19.35,1.76_=50.9; *P*<.001), but not of their interaction (cue type × presentation angle) (*F*_99,9_=0.469; *P*=.89).

Post-hoc analyses concerning the main effects of cue type (ie, irrespective of presentation angle) revealed a significantly better performance for auditory or combined cues but not for tactile cues compared with no cue. Furthermore, auditory cues were better than tactile cues.

#### Performance: Subgroups

**Figure 6:**
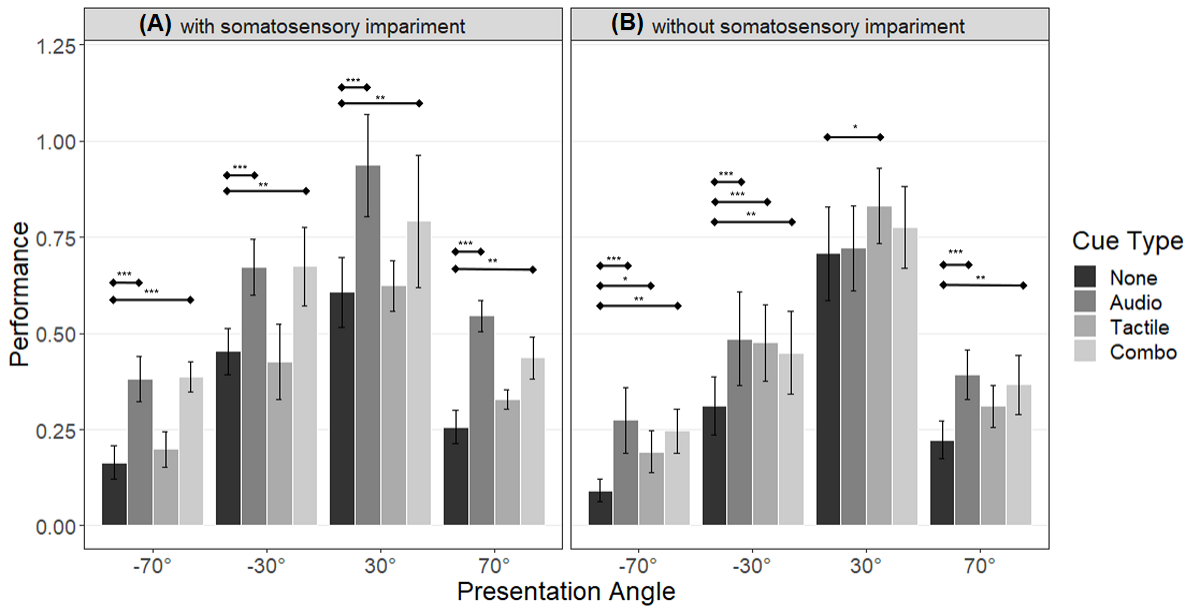
Visualization of the performance, split for the group with somatosensory impairment (A; n=5) and the group without somatosensory impairment (B; n=7). Results of the post-hoc tests are shown as significance bars (level of significance: *P<.05, **P<.01, ***P<.001). The whiskers represent the standard error of means.

Detailed examination of the results (Figure 6) included a 3-way mixed model ANOVA that showed significant main effects of cue type (*F*_30,3_=9.863; *P*<.001) and presentation angle (*F*_17.24,1.72_=47.609; *P*<.001), and a significant 3-way interaction between cue type, presentation angle, and group (*F*_90,9_=2.057; *P*=.04). There were no other significant interactions.

The post-hoc analysis revealed that for patients without somatosensory impairment, any cue led to better performance than no cue for targets on the left side. In patients with somatosensory impairment, performance was better with both auditory and audio-tactile cueing than with no cue, at every presentation angle; conversely, tactile cueing alone had no significant effect at any presentation angle.

#### Early Orientation: All Patients

**Figure 7:**
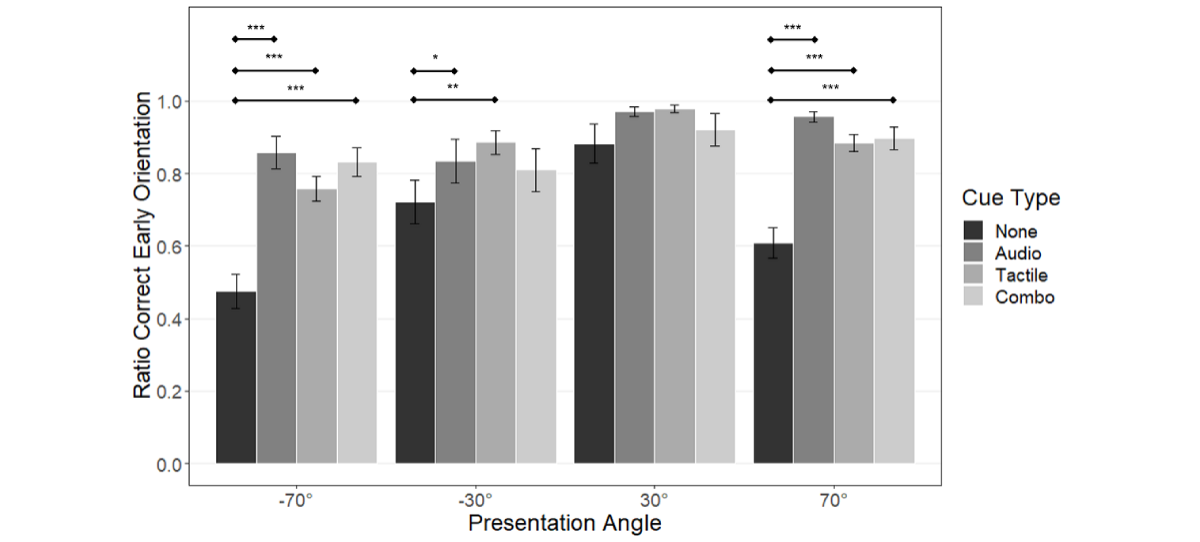
Visualization of the ratio of correct early orientation instances. The whiskers represent the standard error of means. Results of the post-hoc tests are shown as significance bars. The asterisks above the bars represent the level of significance (*P<.05, **P<.01, ***P<.001).

Results of the ratio of correct early orientation for all patients are shown in Figure 7. Repeated measures ANOVA showed significant main effects of cue type (*F*_36,3_=26.934; *P*<.001), presentation angle (*F*_36,3_=10.207; *P*<.001), and their interaction (*F*_99,9_=4.798; *P*=.001). Having cues at lateral angles led to an improvement in the ratio of correct early orientation. Comparison of the same cues at different presentation angles showed that in the None and Audio conditions, there was a significant left-right difference for the paracentral (None: *P*=.002; Audio: *P*=.008) and lateral (None: *P*=.008; Audio: *P*=.047) angles. This pattern was not observed in the Tactile and Combo conditions, where only the lateral (Tactile: *P*=.01) and paracentral (Combo: *P*=.03) angles differed significantly. The significance bars for direct cue comparisons are not shown in Figure 7.

#### Early Orientation: Subgroups

A mixed model ANOVA for cue type, presentation angle, and group showed significant main effects of cue type (*F*_30,3_=32.671; *P*<.001) and presentation angle (*F*_30,3_=13.97; *P*<.001), as well as the following 2 significant 2-way interactions: group × presentation angle (*F*_30,3_=6.738; *P*=.001) and cue type × presentation angle (*F*_90,9_=4.412; *P*<.001). No other interaction reached significance.

**Figure 8:**
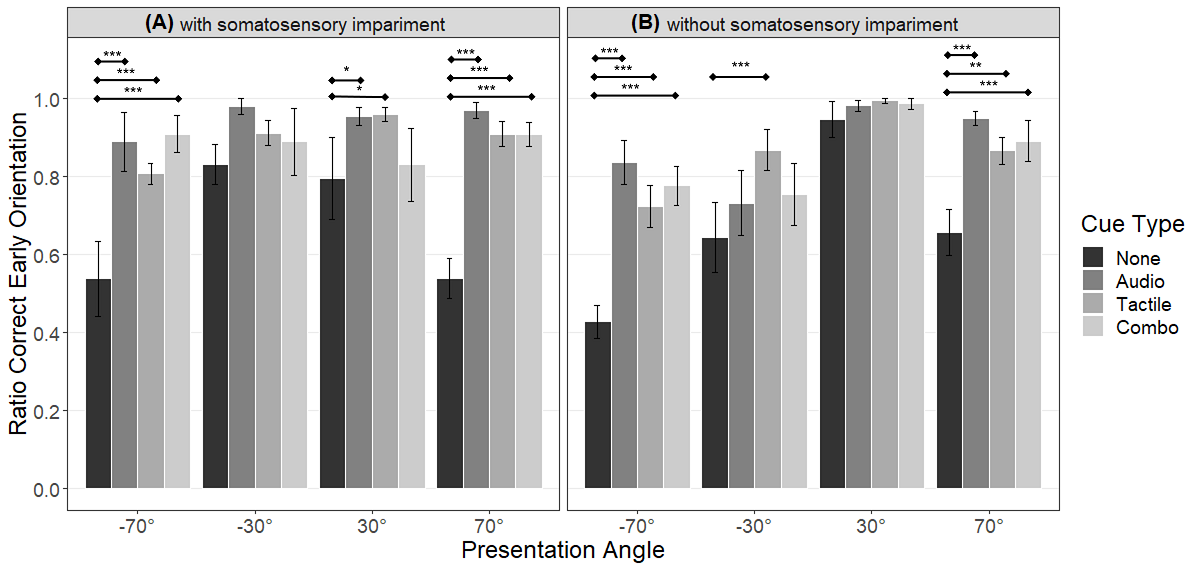
Visualization of the interaction result of presentation angle and cue type, split for the group with somatosensory impairment (A) and the group without somatosensory impairment (B). The whiskers represent the standard error of means. Results of the post-hoc tests are shown as significance bars. The asterisks above the bars represent the level of significance (*P<.05, **P<.01, ***P<.001).

The lack of a significant 3-way interaction also showed that any type of cue (auditory, tactile, or combo) triggered better orientation in both groups for lateral angles (Figure 8).

The significant interaction between group and presentation angle showed a significant difference between the groups for left paracentral angles and a left-right difference for lateral and paracentral angles in the group without somatosensory impairment.

## Discussion

### Overview

This study showed that our new multimodal VR setup, including visual, auditory, and tactile cues, is highly usable and that it is suitable to provide multimodal spatial cues to patients who have spatial attention deficits. Furthermore, the measurements provided some important information concerning the peculiarities of the application of such a setup in this population. Indeed, all patients seemed to benefit from auditory cues; however, the positive effect of tactile cues on in-task performance seemed to depend on the presence of somatosensory impairment. Patients without somatosensory impairment did benefit from tactile cues. Nevertheless, there seemed to be a consistent alerting effect for all cue conditions (auditory, tactile, and combined) among all patients, which could help them to overcome the initial tendency to orient toward the right, a common symptom observed in neglect. Finally, we did not find an additive effect of combining auditory and tactile cues in any measure or group.

### Usability

In line with our first hypothesis, patients rated the device’s usability as very high and did not report any relevant side effects. The high usability of our new VR setting, including tactile stimulation, is similar to that in one of the few previous studies in stroke patients with a similar visual and auditory setting, which however did not entail any tactile stimulation [14,37]. Moreover, similar levels of usability have been reported by healthy participants using new VR tools including visual and auditory stimulation, but without tactile stimulation [36,50,51]. Hence, in our study, the additional presentation of tactile stimulation did not change usability or cause discomfort. Interestingly, in our last study in healthy participants [29], tactile stimulation, similar to that presented here, caused some side effects. We hypothesize that one reason for the minor discomfort caused by tactile stimulation is the duration of application of the tactile stimulation. Indeed, in our previous study, tactile stimulation was continuously presented over several minutes, whereas in this study, it was only presented during short intervals (ie, up to a maximum of 4 seconds per target). This study and our previous study [29] thus represent in some way maximum and minimum usage of tactile stimulation. Future studies should therefore target an optimal tactile stimulation time for maximum effectiveness, but without side effects.

### In-Task Parameters

Our second hypothesis concerned in-task parameters and assumed that patients with spatial attention disorder would benefit from spatial cues, as assessed by means of a compound performance measure, considering both search time and percentage of found targets. Usually, those cues are presented on a 2D screen with a limited visual angle. The advantage of our VR approach is that we can present the patients with far more lateral cues in a virtual environment, supporting them in orienting toward the left by means of not only eye movements, but also head movements. Even though some tools have been developed and tested to examine the effects of visual, auditory, or even tactile cues in patients with impaired spatial attention [21,52], to the best of our knowledge, so far, no study has examined the effects of tactile cues in neglect patients using a VR system.

The auditory cueing of our VR setup was effective in all patients, increasing performance and confirming our hypothesis. The new approach with tactile cueing in VR was also effective, as long as patients had preserved somatosensory processing. This is in line with the principles of unimodal or multimodal cueing, a common approach to guide the attention of patients with spatial attention disorders toward the neglected side [24,53,54].

The fact that tactile cueing can induce positive effects in patients without somatosensory impairment is particularly remarkable. Indeed, tactile cues were presented at different locations in space than visual stimuli; the tactile cues were presented on the skin of the forehead (ie, within the personal space), whereas visual targets were presented in the peripersonal space. This configuration is different from the one concerning auditory cues, were visual targets and auditory cues are both presented at the same location in the peripersonal space [55,56]. In this group of patients, this suggests the activation of spatial attention networks through not only spared supramodal mechanisms [52,57] but also “supraspatial” (ie, entailing different spatial reference frames) mechanisms.

Our results seem to not provide support for the third hypothesis, that is, an advantage of the combined application of tactile and auditory cues in comparison with unimodal cues, since this did not lead to a significant increase in performance or a higher rate of correct early orientation. Some evidence in the literature shows that the use of multimodal cues can have an additive effect [29,31], whereas other studies have shown no benefit of using multimodal cues when compared with unimodal cues [54]. A possible explanation for these disparities could be associated with the level of spatiotemporal matching [58,59] and cross-modal intensity matching [60] needed for cross-modal cues to have an additive effect. In our study, auditory cues had a high spatiotemporal match with the visual target, but tactile cues did not [54]. This might be even more relevant for neglect patients, as they typically show problems with spatial transformations [61-63].

Besides performance parameters (eg, search time and percentage of found targets), we also assessed early orientation behavior. Interestingly, in this case, all types of cues (auditory, tactile, and combination) had positive effects on early orientation, even in patients who had a somatosensory impairment and could not correctly localize a tactile cue. This might be explained through a general alerting effect, as it has been shown that a temporary alertness enhancement can ameliorate attentional orienting in neglect patients [64-66], but this effect is short lived [67,68]. Therefore, it seems reasonable to hypothesize that all types of cues can ameliorate early orientation through their alerting effect and that this effect is not long enough to further support performance to find targets.

### Limitations

In this pilot study, we aimed to show the feasibility and usability of our VR setup in a diverse and complex patient group. On the one hand, the variance increases the difficulty of the interpretability of the task results, and on the other hand, the variety increases the representativeness for this patient group. Neglect severity shows spontaneous fluctuations and intraday variations [69]. We tried to minimize this effect by having the 2 sessions on consecutive days and at the same time; nevertheless, factors like medication changes and exhausting therapies beforehand could not be controlled. Future studies with a larger sample size should try to control or at least assess such confounders

### Conclusion and Outlook

This study showed the usability and feasibility of a new approach entailing auditory, tactile, and combined audio-tactile cueing in VR among patients with different combinations of attentional and somatosensory impairments. Our data suggest that auditory and tactile cues may be equally efficient in ameliorating attentional performance in neglect patients, at least in those with spared somatosensory processing. Moreover, combined audio-tactile stimulation did not show an additive effect in our setup. Future studies are needed to assess these preliminary findings in a larger group of patients. One of the possible directions to reach intermodal additive effects would be to assess the effects of tactile cues presented “nearer” to the source of the visual and auditory stimuli (eg, tactile stimulation on the hand, with variation in presentation side and intensity according to how near the patient moves the hand to the target).

Overall, audio-tactile cueing seems to be a promising method to guide patient attention. For instance, in the future, it could be used as an add-on approach that supports attentional orientation during established therapeutic approaches (eg, optokinetic stimulation).

## Abbreviations

CBS: Catherine Bergego Scale
CoC: Center of Cancellation
HMD: head-mounted display
SNT: Sensitive Neglect Test
SSQ: Simulator Sickness Questionnaire
SUS: System Usability Scale
VR: virtual reality
